# Avoiding transduction-induced heating in suspended microchannel resonators using piezoelectricity

**DOI:** 10.1038/s41378-021-00254-1

**Published:** 2021-04-29

**Authors:** Damien Maillard, Annalisa De Pastina, Amir Musa Abazari, Luis Guillermo Villanueva

**Affiliations:** 1 Advanced NEMS Laboratory, Institute of Mechanical Engineering, École Polytechnique Fédérale de Lausanne (EPFL), 1015 Lausanne, Switzerland; 2 Center for Research on Adaptive Nanostructures and Nanodevices (CRANN), Trinity College Dublin (TCD), Dublin 2, Ireland; 3 Department of Mechanical Engineering, Faculty of Engineering, Urmia University, Urmia, Iran

**Keywords:** Sensors, Electrical and electronic engineering, Biosensors

## Abstract

Calorimetry of single biological entities remains elusive. Suspended microchannel resonators (SMRs) offer excellent performance for real-time detection of various analytes and could hold the key to unlocking pico-calorimetry experiments. However, the typical readout techniques for SMRs are optical-based, and significant heat is dissipated in the sensor, altering the measurement and worsening the frequency noise. In this manuscript, we demonstrate for the first time full on-chip piezoelectric transduction of SMRs on which we focus a laser Doppler vibrometer to analyze its effect. We demonstrate that suddenly applying the laser to a water-filled SMR causes a resonance frequency shift, which we attribute to a local increase in temperature. When the procedure is repeated at increasing flow rates, the resonance frequency shift diminishes, indicating that convection plays an important role in cooling down the device and dissipating the heat induced by the laser. We also show that the frequency stability of the device is degraded by the laser source. In comparison to an optical readout scheme, a low-dissipative transduction method such as piezoelectricity shows greater potential to capture the thermal properties of single entities.

## Introduction

Micro- and nanoelectromechanical systems (M/NEMS) have long been established as physical sensors. While such devices can be operated in static mode through surface-stress effects^1,2^, their operation as resonators in dynamic mode offers a wider range of applications^3^. In this latter configuration, the magnitude of interest is the resonance frequency of the device, and the performance and stability are better when the mechanical losses are low (high quality factor). The great promise shown by resonant beam sensors has rapidly attracted interest for applications in the biological field^4^. Nevertheless, the study of biological samples typically implies a liquid environment. Immersing the resonant beams in fluid leads to a degradation of the quality factor due to viscous drag, especially for flexural beams^5^.

An elegant solution to this issue was brought to the microscale by Burg and Manalis^6^, where a microfluidic channel was made part of a resonant beam^6^. These so-called suspended microchannel resonators (SMRs) were later encapsulated in vacuum, showing quality factors up to three orders of magnitude higher than the same resonator immersed in liquid^7^. Therefore, the use of SMRs reduces damping, and thus one can reach better sensitivities^8^. Over the years, applications of SMRs have diversified: measurement of the pressure^9^, density^10,11^, and viscosity^12^ of homogeneous samples or characterization of the mass, density, volume, growth rate, and deformation of populations of cells^13,14^.

A field that remains elusive is the calorimetry study of single biological entities. Typically, biocalorimetry experiments study tissue aggregates or multiple analytes together^15,16^. The community indeed lacks tools to measure the thermal properties of single biological entities, which have been reported only a few times^17–19^. SMRs are a great candidate to become such a tool.

However, to exploit the full sensing potential of these devices, the transduction strategy needs to be optimized. Most of the SMRs presented in the literature are actuated with a piezo-ceramic shaker^12,13^ or, in some cases, electrostatically^6,20^. On the one hand, the latter option can be implemented on a chip, but then the device cannot operate over its full dynamic range (either weak actuation or strong nonlinearity). On the other hand, using a shaker requires attaching the chip to the piezo-ceramic slab, inevitably making the system bulkier. We recently showed the piezoelectric (PZE) actuation of SMRs, which is integrated on-chip, is linear, and allows for large amplitudes to be reached^21^.

Regarding detection, the most commonly used measurement methods are optical-based, whether they consist of optical levers^6^, interferometers^10^, or laser Doppler vibrometers^9,12,22^. Although they offer excellent performance, those solutions are bulky, generally costly, and time-consuming to set up. In addition, a portion of the laser power is absorbed by the SMR; therefore, heating the SMR and shifting the resonance frequency. This effect strongly depends on the position of the laser during measurement and the laser power fluctuations due to heat-induced local stresses at the laser position^23,24^. For this reason, some groups have developed optical-free readout methods, such as piezoresistive^25,26^, PZE through quartz tuning fork coupling^27^, or electrostatic^11^ methods. A more exhaustive list of the existing transduction techniques for SMRs can be found in our recently published review^28^.

In this manuscript, we present for the first time full-on-chip PZE transduction of flexural SMRs with integrated electrodes. This transduction mechanism offers notable advantages; for example, the electrodes are directly integrated on the chip, and the operation of the devices dissipates little power. Our experimental setup allows us to compare the measurement noise between PZE transduction and an alternative detection scheme^29^. Indeed, we show that focusing a laser source on the beam creates a shift in the resonance frequency due to the local temperature increase of the SMR. This effect is modulated by the flow rate of the liquid within the microfluidic channel: a higher flow rate accelerates the cooling of the SMR and reduces the heat-induced frequency shift. We also compare the frequency stability in different situations and show that focusing the laser on the device significantly degrades its performance. We show that for our devices, avoiding the use of heat absorption within the transduction is preferential to reach better frequency stability and, as such, improve the sensitivity of the resonators. PZE SMRs also show potential for pico-calorimetry applications.

## Results and discussion

It is well known that the resonance frequency of SMRs depends on their effective mass and thus varies with the density of the fluid flowing inside the channels^10,11,21^. The temperature behavior of such devices as operated with PZE actuation and optical readout, empty and fluid-filled, was previously described^29^. However, in that former experiment, the readout laser heated the SMRs and thus affected the operating temperature of the device. With full PZE transduction, we can remove the laser-heating effect and have more control over the experiment. We do this by encapsulating the devices in vacuum in a dedicated interface and by then sweeping the temperature of the chip between 25 and 50 °C. Each temperature set point is stabilized by a thermoelectric temperature controller (ILX Lightwave LDT-5910C, Newport, USA), implemented with proportional integral derivative (PID) control. The controller reads the temperature from a thermistor located inside the metallic fluidic connector and consequently adjusts the current input of a Peltier module in direct contact with the interface. After changing each set point, we wait for ~5 min for the temperature to be uniform on the chip. The frequency is monitored with a phase-locked loop (PLL) control from a lock-in amplifier (UHFLI, Zurich Instruments, Switzerland).

**Fig. 1:**
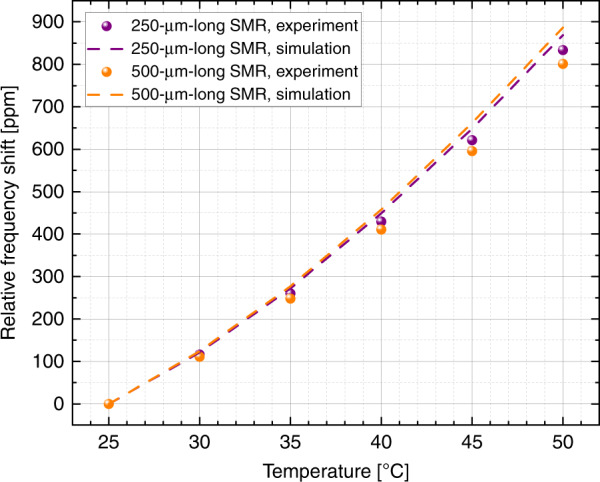
Frequency dependence of SMRs on uniform temperature. Resonance frequency of the first mode of a 250-μm- and a 500-μm-long SMR filled with deionized water, as a function of temperature applied using uniform heating. Even though the Young’s modulus of the structural material decreases with decreasing temperature, making the device more compliant, the resonance frequency change is dominated by the decreasing density of the water

Figure 1 shows the relative frequency shifts of a 250-μm- and a 500-μm-long SMR filled with deionized water, measured with full PZE transduction. As expected, when the temperature increases, the resonance frequency of the SMR follows the same trend. Indeed, the density of the water is inversely proportional to the temperature and decreases at a faster rate than the Young’s modulus of the resonator’s structural material. It is notable that the data are valid for any SMR length. The responsivity of the device with respect to the temperature only depends on the ratio of the cross-sectional areas of the solid and the fluid of the SMR^29^. Figure 1 also shows great agreement in the comparison of our experimental data to a finite-element analysis simulation without fitting parameters.

Following this initial characterization, we investigate the heat that is transferred to the filled SMR from our optical detection system, consisting of a laser Doppler vibrometer (LDV OFV-5000 with OFV-551, Polytec, Germany). This allows us to estimate how much the laser readout affects the previously published results^29^. To do this, we focus the laser at the tip of the SMR and switch it on and off repeatedly and for different periods of time (5, 10, and 20 s) while tracking the frequency. The laser power was determined independently with a power meter (PM100D, ThorLabs, USA) and was ~645 μW.

**Fig. 2:**
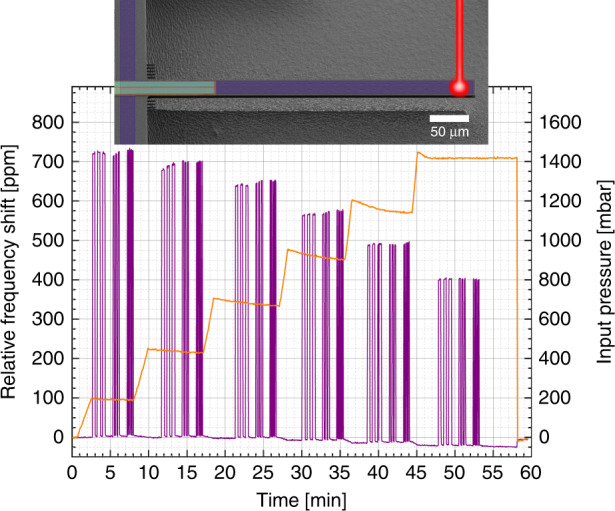
Laser-heating effect on the SMR frequency. While the resonance frequency of the SMR is continuously detected via piezoelectric transduction, the laser beam is focused on the tip of the beam and switched on and off repeatedly. We observe a positive frequency shift when the laser turns on and a recovery of the baseline after shut-off. The magnitude of the shift does not depend on the illumination time. The experiment is performed at different input pressures and lasts ~1 h

We repeat this procedure at different input pressures within the microfluidic channel, as shown in Fig. 2. The liquid pressure is measured by a pressure sensor on the fluidic line before the connection to the interface. The pressure is applied using a syringe pump (Low-Pressure module, CETONI GmbH) controlled by a PID loop operated through dedicated commercial software (Qmix Elements, CETONI GmbH).

As shown in Fig. 2, when the beam collides with the SMR, a positive shift in resonance frequency is immediately detected. This effect looks similar to that of a uniform increase of 20 °C in the temperature of the system, if compared to Fig. 1. The resonance frequency reverts back to its original value as soon as the laser beam is switched off. We do not acknowledge any significant difference in the resonance frequency shifts depending on how fast the laser is switched on and off and how long it is kept on. We attribute this behavior to a temporary and local change in the water density as it is heated up by the laser. As the laser shuts off, the flow of water inside the SMR carries away excessive heat, and the system returns to the initial conditions.

As we increase the fluidic pressure, we notice two things: (i) a reduction in the frequency baseline, which is attributed to the resonance frequency dependence on pressure^9^ and (ii) a reduction in the shift (Fig. 2) due to laser illumination. This latter effect is a clear evidence that the water acts as a cooling medium via convection and that it is possible to enhance the cooling efficiency through a higher flow rate.

The first effect shows a reduction in the baseline frequency between the beginning of the experiment at 0 bar pressure and the end at ~1.5 bar. We can characterize the pressure responsivity of our SMR: −24 p.p.m./bar. Our understanding is that the input pressure creates an expansion of the channel volume, leading to an increased effective mass. Since the frequency shift is negative, it seems that this effect dominates over the stiffening of the beam. Nevertheless, this effect is negligible when compared to the effect of laser illumination.

**Fig. 3:**
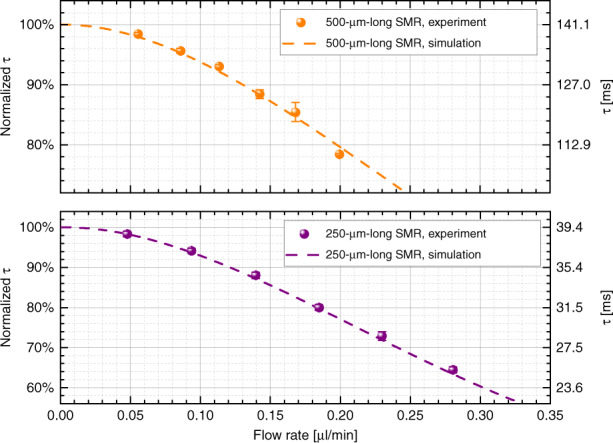
Thermal time constant dependence on the flow. The time constant *τ* of the frequency transition when the laser is switched on is studied for both devices. The time constant is decreasing with increasing flow rate, confirming that higher fluid velocity in the channel is more efficient to cool down the SMR. The two devices behave similarly, with the absolute time constant of the 500-μm-long SMR being 3.5 to 3.75 times higher than that of the 250-μm-long SMR (*τ* scales with the square of the length)

In this paper, we present experiments that use SMRs of two different lengths (250-µm- and 500-µm-long) found on different chips, each of which has different channel lengths. In particular, the 250-µm-long SMR (device A) is part of a channel with two resonators, while the 500-µm-long SMR (device B) is part of a channel with six resonators. Therefore, the fluidic resistances of the microchannels are different, and the same pressure does not provide the same flow rate in each of the chips. To appropriately compare the effect of laser heating and convection cooling on the different devices, we plot the measurements with respect to the flow rate instead of the input pressure, which is experimentally fixed, since the measurements are performed in a pressure-controlled setup. To appropriately calculate the flow rate, we first divide the applied pressure at the inlet by the fluidic resistance of the respective channel. According to their geometrical dimensions, the channel of device A features a fluidic resistance of ~5.26 bar/(µl/min), whereas the channel of device B has a resistance ~1.46 times higher due to the additional length. To determine the dimensions of each channel, we used the experimentally determined values after cutting through an SMR via focused ion beam technology and observed its cross-sectional dimensions with a scanning electron microscope. The details of the measurements are available in the Supplementary information. To fine-tune the flow rates, we use the results of finite-element modeling (FEM) transient simulations of the stabilization of temperature in our system compared to experimental results. Benefiting from our PLL tracking of the frequency, we can study the time constant of the transition. Looking closer at the apparently abrupt changes in frequency that are seen in Fig. 2, we can indeed see exponential decay behavior, which can be fitted to extract the time constant for each event (see Supplementary information). Figure 3 shows the extracted time constants vs flow rate, both in absolute and relative terms. Importantly, the values of these time constants are dependent on the thermal conductivity, cross-sectional dimensions, and flow rate. Thus, it is possible to use the comparison between the experiment and simulation to (i) determine that the thermal conductivity of our silicon nitride is *κ*_SiN_ ≈ 4 W/(mK) and (ii) fine-tune the values of the fluidic resistances mentioned above, which are *R*_f,250 μm_ = 5.26 bar/(µl/min) (same as estimated from dimensions) and *R*_f,500 μm_ = 8.63 bar/(µl/min) (12% larger than estimated from dimensions).

The behavior shown in Fig. 3 can be explained considering three sources of heat dissipation in the system: (i) conduction through the ls-SiN_*x*_, (ii) conduction through the water, and (iii) convection in the water due to the flow. As the devices are operated in vacuum (0.01 Pa pressure), convection and conduction to the air around the device are neglected. We also neglect the effect of thermal radiation because the temperature changes are not very large (see further discussion below). We notice that the FEM results show a planarization of the value of the thermal response time for flow rates below 20 nl/min (Fig. 3). At lower flow rate values, conduction dominates, and thus no dependence on the flow rate is seen. For the range of flow rate values that we use in experiments, convection due to the internal liquid is the dominant mechanism for heat transfer.

**Fig. 4:**
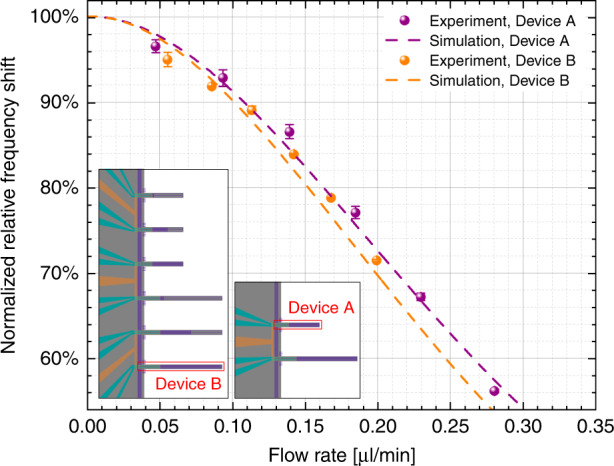
Cooling effect of internal liquid. Effect of fluidic flow on the local heating induced by the laser focused on the cantilever’s tip. Each point represents the relative frequency shift caused by a change in laser state (on/off), normalized to the maximum shift (with no flow rate). Experiment data and simulations are shown for two devices of two different chip configurations. There is a decrease in the frequency shift with respect to the flow rate, illustrating a more efficient cooling of the device as the fluid velocity increases. Insets: Device A has a length of 250 μm and is part of a 2-SMR array, while Device B extends for 500 μm, being part of a more complex 6-SMR array

Using the adjusted values for the fluidic resistances, we can also plot the relative frequency shift for each SMR under study as a function of the flow rate, which we show in Fig. 4. We overlap the experimental results (scattered data, with error bars) with the results from an FEM simulation using modal analysis with the stationary thermal state as a boundary condition. The matching between the FEM and experimental results is remarkable. We can see how for flow rates <20 nl/min, the simulated response flattens out, as was the case for the thermal response times. For larger flow rates, the frequency shift decreases in magnitude when the flow rate increases. For example, in device A, a flow rate of 280 nl/min corresponds to a frequency 44% lower than in the case of no flow rate. As discussed above, the flow rates for each device are different because the fluidic resistances are different. For device B, it is not possible to reach higher flow rates because otherwise, the required input pressure could break the inlet membrane.

**Fig. 5:**
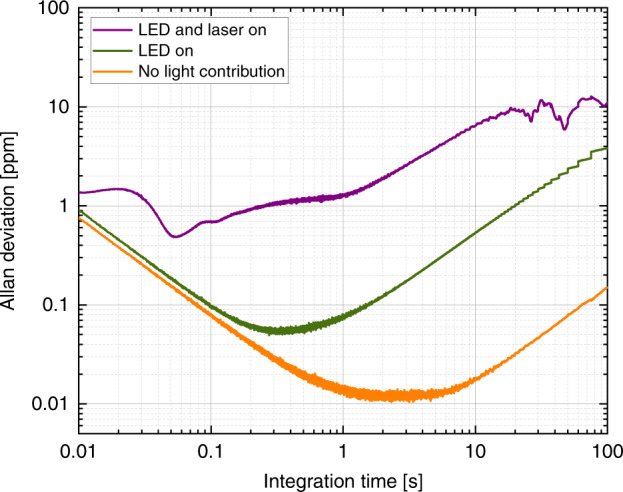
Effect of illumination on frequency stability. Allan deviation of a 500-μm-long SMR with water flowing at ~50 nl/min (input pressure 370 mbar). A minimum deviation below 20 p.p.b. with integration times between 2 and 4 s is achieved when all external light contributions are removed. In contrast, the LED (for cantilever imaging) and the laser contribute significantly to the noise of the resonator

The last interesting question that remains is whether the laser-heating effect, which affects the value of the resonance frequency, also affects our ability to determine this frequency. To evaluate that, we measure the frequency stability and calculate the Allan deviation, which is directly proportional to the sensitivity of a given resonating sensor and is the recognized tool to assess device performance in the M/NEMS community^30^. For this experiment, we keep the flow rate at a constant value of ~50 nl/min, and we track the resonance frequency with a PLL (bandwidth 100 Hz). We recorded the frequency for 5 min under three distinct conditions: without any external light contribution, with the standard white light for microscope illumination focused on the beam, and with both the white light and the LDV laser focused. Figure 5 depicts the Allan deviation in the three different cases. We notice that additional light contributions are detrimental to the device performance. In particular, switching on the LDV detection scheme worsens the stability by two orders of magnitude (with an integration time of 1 s). This result indicates that the performance of SMRs is greatly reduced when an optical readout is implemented. To reach ultimate levels of detection, it seems that adopting on-chip, built-in readout techniques, such as piezoresistive or PZE techniques, would yield higher sensitivities.

In this manuscript, we demonstrate for the first time full on-chip PZE transduction of flexural SMRs. This detection scheme allows us to analyze the back-action of an alternative and widely used optical detection method—laser Doppler vibrometry—on our device. We show that the laser beam brings a significant amount of heat, measured with the resonance frequency shift, to the SMR. The extent of the frequency shift can be controlled by the flow rate of the fluidic analyte, indicating that convection is a significant mechanism of heat dissipation. We even observe that a significant improvement in frequency noise is attained when no external light illuminates the SMR.

Although on-chip PZE transduction increases the complexity of the fabrication and adds to the effective mass of the SMR, the advantages outweigh the drawbacks since we gain frequency stability (compensating for the increase in effective mass) and simplify the overall experimental setup, which can mean a paradigm shift for the field of calorimetry of single cells.

## Materials and methods

**Fig. 6:**
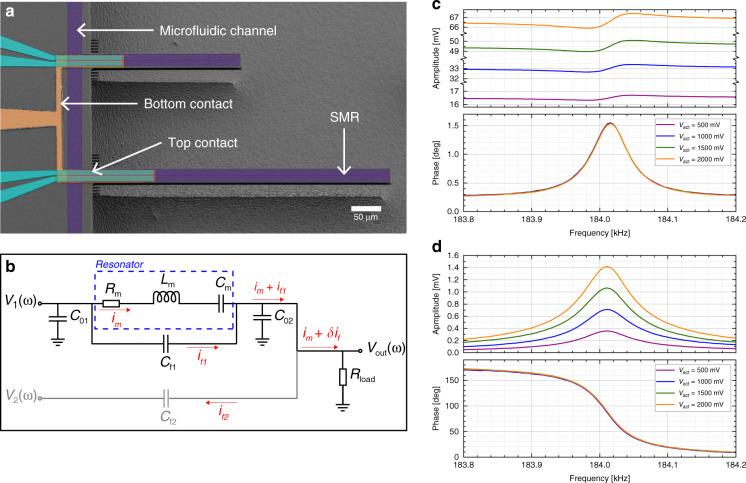
SMRs with piezoelectric transduction. **a** Colored scanning electron microscope image of an array of two SMRs, 250 and 500 μm long, with their integrated PZE electrodes on top. The microfluidic channel is represented in purple, the bottom contact in orange, and the top contacts in cyan. **b** Electrical drawing of the balancing bridge circuit implemented to lower the level of parasitic background. **c** Frequency sweep of the amplitude and phase of a 250-μm-long SMR filled with deionized water without balancing circuit, for different actuation voltages. The signal-to-background ratio is ~1.012. **d** Same frequency sweep using the balanced bridge configuration. The signal-to-background ratio is greatly enhanced and >30

The structural material of the PZE SMRs used in this work is low-stress silicon nitride (ls-SiN_*x*_). The devices consist of singly-clamped beams with cross-sectional dimensions of ~7 × 30 μm^2^ and lengths of 250 or 500 μm. From inlet to outlet, the microfluidic channel runs through several SMRs located in series with the channel, but physically organized in parallel (see Fig. 6a). In each resonator, the embedded channel forms a U-turn at the tip of the beam, and its dimensions are ~10 ×5 μm^2^. More accurate dimensions can be found in the Supplementary information. As shown in Fig. 6a, each resonator features a 25-nm-thick platinum ground electrode. A 300-nm-thick layer of PZE material, aluminum nitride, was deposited and covered with another layer of platinum (50 nm) forming the top electrode. These two layers were subsequently patterned, creating two separate fingers for independent actuation and detection of the SMRs. More details about the fabrication of those devices can be found elsewhere^21,31^. After the wafer was cleaved, the chips were interfaced and encapsulated by a custom-made microfluidic connector also serving as a vacuum chamber^29^.

We perform the dynamic characterization of our devices using the Zurich Instrument lock-in amplifier. We start by sending a harmonic signal to one top electrode while recording the output signal from the other top electrode (their ground is common), and we do this while sweeping the signal’s frequency around the resonance frequency of the device. Figure 6c shows frequency sweeps of the PZE signal measured at the detection electrode for actuation voltages from 500 mV to 2 V. We can see that the resonance peak is buried in a large background; the linear increase of the signal level away from resonance as well as the small phase change across the resonance are problematic for suitable detection. This large background level is due to the design and fabrication choices: the electrode tracks cover a large area on the wafer, and the polysilicon layer used as a sacrificial material to fabricate hollow channels is heavily doped with conducting POCl_3_. Both points contribute to a very large parasitic feedthrough capacitance. A detailed electrical layout depicts the situation in Fig. 6b (in black), where *C*_f1_ represents the feedthrough capacitance between the actuation and detection electrodes. Within this schematic, the motional current coming from the SMR (*i*_m_) is superimposed with a parasitic current *i*_f1_, which is directly proportional to the magnitude of *C*_f1_. Since the inherent linearity of PZE detection makes it difficult to implement most of the methods for background cancellation^32,33^, we decide to balance, away from resonance, the current at the output point. This strategy has been used in the past for different transduction techniques^34–38^. We implement it by connecting a second resonator on the same chip, but operating at a different resonance frequency with respect to the device to detect. We feed this second device a harmonic signal at the same frequency and amplitude as the driving signal, but with a phase shift of ~180°. By doing this (in gray in Fig. 6b), we remove most of the background from the output point. This can be seen in detail in Fig. 6b (in gray), where a second parasitic current *i*_f2_ reduces the background from the output point. By carefully tweaking the amplitude and phase of *V*_2_(*ω*), we can reduce the contribution of *i*_f1_ to the output current of the resonator of interest by more than three orders of magnitude. Therefore, we can see a much higher signal-to-background ratio than in the unbalanced configuration, as is shown in Fig. 6d. The amplitude now portrays a resonance peak standing well above the background, and the phase undergoes a 180° phase shift; these characteristics allow the device to be operated within PLL to easily track the resonance frequency over extended periods of time.

## Supplementary information

Supplementary information
