# Supplementary material for: Avoiding transduction-induced heating in suspended microchannel resonators using piezoelectricity
Source: Microsyst Nanoeng. 2021 Apr 29;7:34. doi: 10.1038/s41378-021-00254-1 (PMC8433141; doi:10.1038/s41378-021-00254-1)
Supplement: Supplementary file 1 — Supplementary information [file 41378_2021_254_MOESM1_ESM.docx]

**SUPPLEMENTARY INFORMATION**

Avoiding transduction-induced heating in suspended microchannel resonators using piezoelectricity

Damien Maillard

^1^Advanced NEMS Laboratory, Institute of Mechanical Engineering, École Polytechnique Fédérale de Lausanne (EPFL), 1015 Lausanne, Switzerland

[damien.maillard@epfl.ch](mailto:damien.maillard@epfl.ch)

Annalisa De Pastina

^1^Advanced NEMS Laboratory, Institute of Mechanical Engineering, École Polytechnique Fédérale de Lausanne (EPFL), 1015 Lausanne, Switzerland

^2^Center for Research on Adaptive Nanostructures and Nanodevices (CRANN), Trinity College Dublin (TCD), Dublin 2, Ireland

[depastia@tcd.ie](mailto:depastia@tcd.ie)

Amir Musa Abazari

^3^Department of Mechanical Engineering, Faculty of Engineering, Urmia University, Urmia, Iran

[am.abazari@urmia.ac.ir](mailto:am.abazari@urmia.ac.ir)

Luis Guillermo Villanueva

^1^Advanced NEMS Laboratory, Institute of Mechanical Engineering, École Polytechnique Fédérale de Lausanne (EPFL), 1015 Lausanne, Switzerland

[guillermo.villanueva@epfl.ch](mailto:guillermo.villanueva@epfl.ch)

0041 21 693 11 87

1. FEM simulations

In order to perform FEM simulations as realistic as possible, we choose to create a model with the actual dimensions of the SMR. We cut through the middle of an SMR with Focused Ion Beam technology, and then proceeded to observe the cross-section at the SEM. Fig. S1 depicts a view of the SMR with the dimensions needed to build the model. For each particular feature, we increased the magnification to make a measurement as precise as possible.

| 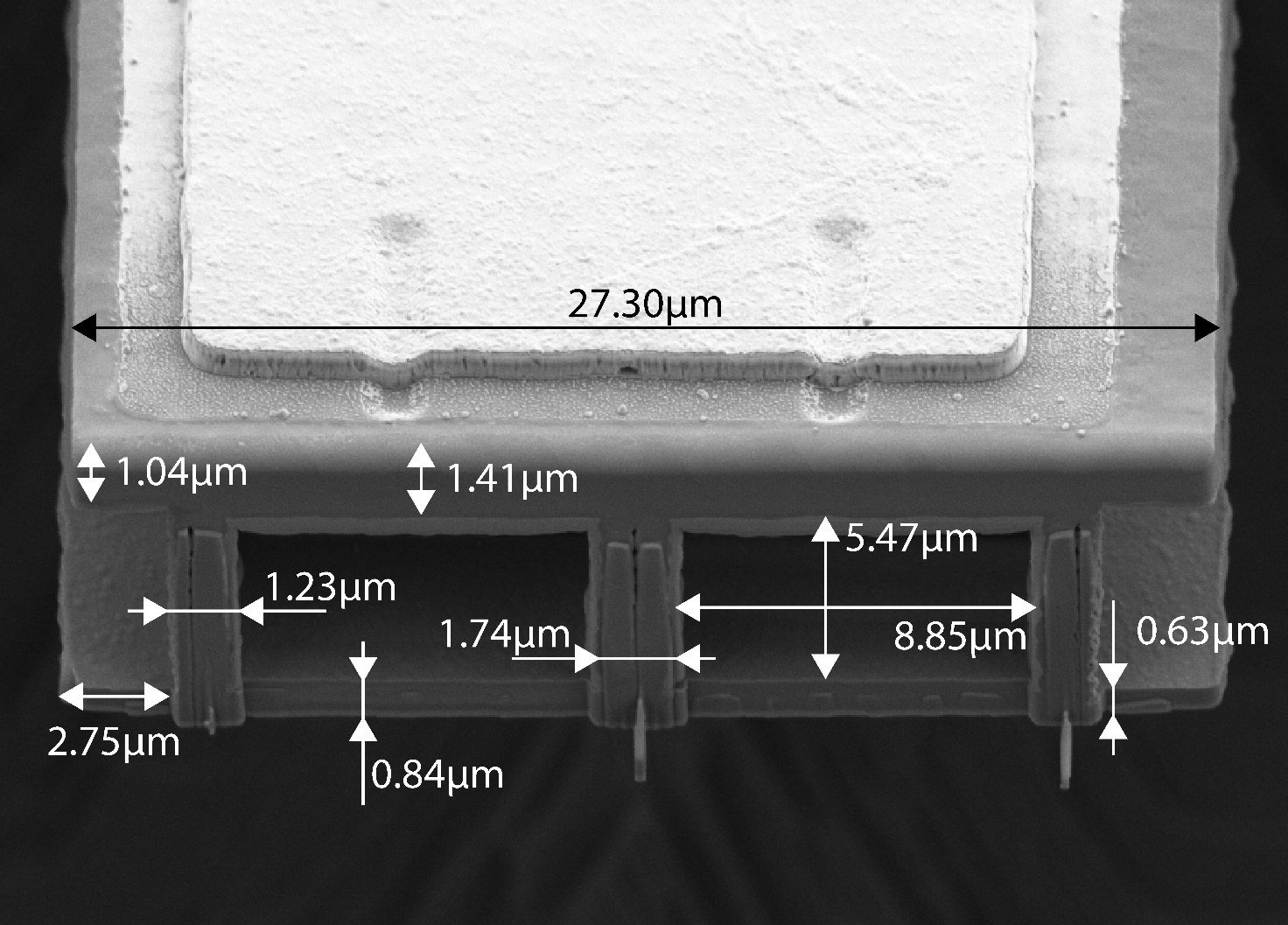 |
| --- |
| Fig. S1: SEM cross-section view of an SMR after cut with FIB. The image is taken on the device while tilted 45°. |

Our model consists of a time-dependent simulation of the evolution of the average temperature of the cantilever (silicon nitride beam and water flowing) when the free end of the cantilever is subject to a heat source. Observing the behavior of the temperature over time for different flow rates allows to extract the $\tau$ of the transition to the stationary state.

In a second step, the temperature results are used in a modal analysis to compute the changes in resonance frequency. After doing this, we can refine the value of the absorbed power and match exactly the frequency jumps we observed experimentally. This power is around 30μW (about 5% of the incident light).

1. Thermal time constant

To extract the time constants of the transition between the two states of resonance frequency when the laser is switched on, we fit the measurement to an exponential decay function, as shown in Fig. S2 for the 250-µm-long SMR.

| 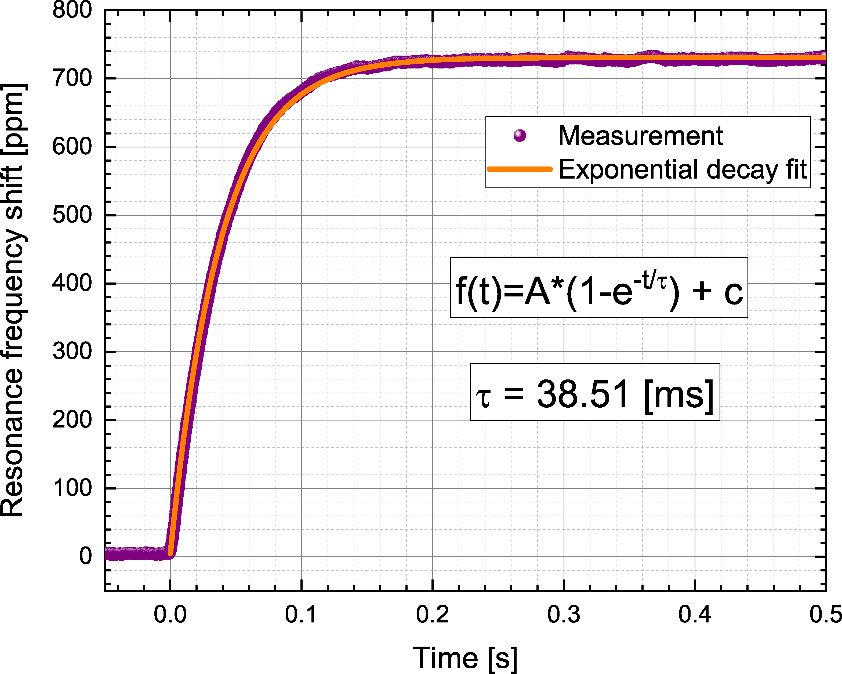 |
| --- |
| Fig. S2: Resonance frequency transition when the laser is switched on. We notice that the data is closely fitted by an exponential decay function, which allows to extract the time constant of the transition. |
